# A Case of *Nocardia kroppenstedtii* Infection Successfully Treated With a Multidisciplinary Approach

**DOI:** 10.1155/crdi/6452173

**Published:** 2025-02-06

**Authors:** Francesco Foglia, Annalisa Ambrosino, Giuseppe Greco, Annalisa Chianese, Carla Zannella, Francesca Cinone, Alfonso Reginelli, Diego Sandro Giordano, Giovanni Tortorella, Maria Teresa Laieta, Anna De Filippis, Massimiliano Galdiero, Rita Greco, Emiliana Finamore

**Affiliations:** ^1^Department of Experimental Medicine, University of Campania “Luigi Vanvitelli”, Naples, Italy; ^2^Department of Advanced Medical and Surgical Sciences, University of Campania “Luigi Vanvitelli”, Naples, Italy; ^3^Department of Precision Medicine, University of Campania Luigi Vanvitelli, Naples, Italy; ^4^UOSD Microbiology, AORN Sant'Anna and San Sebastiano, Caserta, Italy

**Keywords:** antimicrobial diagnostic stewardship, actinomyces infection, *Nocardia kroppenstedtii*, surveillance

## Abstract

*Nocardia* species constitute a diverse group of microorganisms classified as aerobic actinomyces. Among these species, many have been implicated as causative agents of severe human infections, particularly in immunocompromised patients, affecting lungs, skin, and nervous system. Here, we described a rare species, identified as *Nocardia kroppenstedtii,* isolated at the Complex Operative Unit of Virology and Microbiology from the subxiphoid formation and pseudonodular formation in the left leg of a 69-year-old immunocompetent patient, who was hospitalized and treated at the Complex Operative Unit of Internal Medicine and Geriatrics of the University Hospital of Campania “Luigi Vanvitelli” in an antimicrobial diagnostic stewardship context. This rare pathogen was first isolated in 2014 from a bronchoalveolar lavage sample obtained from a lung transplant recipient. Since then, only five cases of clinical interest have been described in literature.

## 1. Introduction

*Nocardia* spp. is a group of Gram-positive bacteria commonly found in soil, water, and organic matter [1]. These bacteria thrive in aerobic conditions and exhibit variable acid-fast staining. The genus was first isolated and described by Edmond Nocard in 1888 [2]. Since then, many species have been identified, thanks to the advent of molecular methods and the increasing availability of whole-genome sequencing [3]. According to the List of Prokaryotic Names with Standing in Nomenclature (LPSN), the *Nocardia* genus currently counts 251 species (https://www.bacterio.net); Among the 251 identified species, only a small subset is pathogenic to humans causing opportunistic and potentially fatal infections, especially in immunocompromised patients like organ transplant recipients, individuals with malignancy and suffering from acquired immunodeficiency syndrome (AIDS) [4].

The clinical picture of nocardiosis depends on the body systems affected by the infection, specifically lungs, skin, and central nervous system (CNS). In 80% of cases, lung involvement occurs due to inhalation, causing severe pneumonia (acute/subacute/chronic infection), which could disseminate to the CNS, evolving in abscesses into the brain and other organs: the onset of skin infections occurs in 20% of cases after a traumatic event through direct skin contact, which could result in lymphocutaneous infection with ulceration of lymph nodes while disseminated infections involving multiple organs are less frequent, and they can be life-threatening [5]. Identifying *Nocardia* species is crucial in clinical practice because their susceptibility to antibiotics is varied [6]. Recently, a fair number of infections occurred in patients with none of the conditions mentioned above, suggesting that a weakened immune system is not the only predisposing factor to infection.

The mechanisms of pathogenesis are not entirely clear and include the ability to evade the host's microbicidal activities. Actually, immunocompetent patients with nocardiosis represent one-third of all cases [7–11].

In the last decade, a new species was isolated from a bronchoalveolar lavage sample obtained from a lung transplant recipient and recognized as *Nocardia kroppenstedtii* in the United Kingdom [12].

Although *Nocardia* species are generally found worldwide, with cases frequently reported in Europe, Asia, North America, and other regions, specific global distribution data for *Nocardia kroppenstedtii* is peculiarly limited, with only few human isolations reported globally. *Nocardia* species, including *Nocardia kroppenstedtii*, are typically associated with soil and water environments, from which they can be transmitted to humans [3].

Here, we present a rare case study involving the isolation of *N. kroppenstedtii* in a patient with a competent immune system treated at the University Hospital of Campania “Luigi Vanvitelli” in Naples, Italy, which highlights the crucial role of the antimicrobial diagnostic stewardship program implemented in our hospital [13–15] in formulating accurate treatment plans.

To the best of our knowledge, this is the sixth reported clinical case in the world.

## 2. Clinical Case Overview

**Figure 1:**
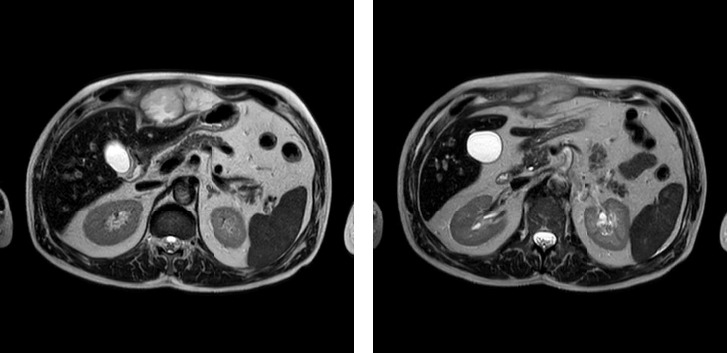
Single-shot fast spin echo T2 weighted magnetic resonance imaging showing a collection in the anterior abdominal wall located at the left paramedian region, which slightly imprints and displaces the left hepatic lobe (a). The content appears mildly and heterogeneously hyperintense on fluid-sensitive sequences, indicating a predominantly fluid component with proteinaceous content. The walls appear moderately thickened. The described finding appears compatible with an abscess collection. Moreover, there is a slight edema and inhomogeneity present in the visceral peritoneal fat (b).

A 69-years-old patient with a mute medical history presented at our hospital with fever, a large subxiphoid-evolving swelling, which developed two weeks after exerting intense effort from a physical activity outdoor, and another lump on the inside of the upper third of the right inner thigh. A thoracic computed tomography (CT) scan performed two weeks before admission, showed inhomogeneous subxiphoid hypodensity and pseudonodular formation in the left leg lobe, with speculated contours and a ground-glass thickening halo; therefore, a therapy consisting of 200 mg cefditoren pivoxil for 10 days and 25 mg deltacortene for a week plus 12,5 mg deltacortene for another week was administered. One week before admission, the patient started to experience fever responsive to paracetamol. Magnetic resonance imaging (MRI) contrast examination showed an increased swelling with irregular and intense thickening and progressive enhancement of the walls with uneven content (Figure 1). The patient was then hospitalized with a blood pressure of 130/60 mmHg, SpO2 of 97%, 38.7°C fever, and a heart rate of 110 BPM. The blood samples showed high neutrophil counts and a CRP 23 times the normal value (10.5 mg/dL). The elevated levels of transaminases, gamma-glutamyl transferase, and alkaline phosphatase indicated a possible compression of the mass on the bile ducts and the liver. The patient was in severe pain, to the extent that he was using opioid patches. A nasopharyngeal swab for SARS-Cov2 detection was done, and the result was negative. Chemiluminescence immunoassay results for Hepatitis B Virus (HBV), Hepatitis C Virus (HCV), and Human Immunodeficiency Virus (HIV) were negative.

The patient underwent a drainage surgical procedure to remove the subxiphoid abscess collection and the pseudonodular formation identified on CT and MRI, with the aim of analyzing and identifying any potential isolates and remained hospitalized in the UOC of Internal Medicine and Geriatrics while the antibiotic treatment was upscaled according to European Society of Clinical Microbiology and Infection Diseases (ESCMID) guidelines to meropenem (3 g/d) and vancomycin (30 mg/kg/d) on the first day.

**Figure 2:**
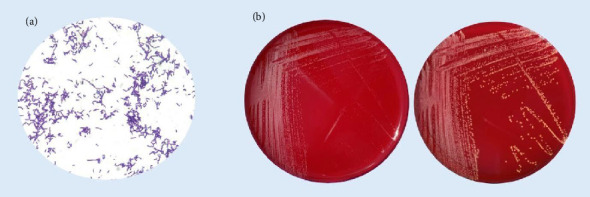
Gram staining performed on drainage fluids at 100X magnification. The staining highlights the filamentous and branched cells of *N. kroppenstedtii* (a)*. N. kroppenstedtii* growing on CNA agar. Note the different morphology after 24 h (on the left), and prolonged incubation (on the right) (b).

The drainage fluids collected during the surgical intervention from the subxiphoid formation and pseudonodular formation were sent at the UOC of Microbiology and Virology, cultured in brain heart infusion (BHI) broth and incubated at 37°C. Meanwhile, the staining of the two samples resulted in Gram-positive bacilli with a branching filamentous morphology that resembled actinomycetes (Figure 2(a)). The next day, the turbidity of the two broths indicated microbial growth. To isolate the eventual infectious agent and both were plated on culture media, including MacConkey agar, Columbia CNA agar with 5% Sheep Blood, Chocolate agar, Sabouraud Dextrose agar (Liofilchem, Italy), and incubated at 37°C for 24 h. After the incubation period, we observed the growth of greyish colonies only on Columbia CNA agar which became yellowish after 4–5 days (Figure 2(b)), for both the fluids. We confirm that the two samples were colonised by the same agent performing matrix-assisted laser desorption ionization-time of flight mass spectrometry (MALDI-TOF MS, Bruker Daltonics, USA) identification. The identification of the microorganism in both isolates was repeated two times for sample and resulted in *Nocardia kroppenstedtii* with a high confidence score.

**Figure 3:**
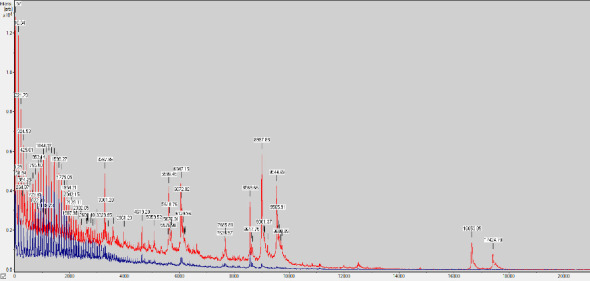
*N. kroppenstedtii* spectrum generated on MALDI-TOF MS.

The data generated by MALDI-TOF MS (Software version 4.1 (100), specific to the species, were analyzed according to the manufacturer's instructions. Additionally, the spectra underwent baseline correction and were adjusted to the total positive ion current [16] (Figure 3).

The antibiotic susceptibility testing revealed that the strain was resistant to meropenem, and high-susceptible to trimethoprim/sulfamethoxazole (TMP-SMX) (Table 1).

**Figure 4:**
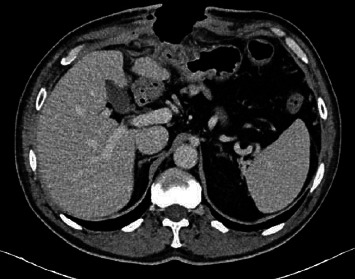
Axial CT portal venous phase section shows a large discontinuity at the level of the anterior abdominal wall, as a result of surgical drainage of the previously noted collection. The postoperative CT revealed a good surgical cleaning of the site, with no further collections observed and no postoperative complications identified.

The identification of *N. kroppenstedtii* with its susceptibility pattern led to an updating of the empirical therapy into a targeted one and IV TMP-SMX (400/80/5 mg) was started at a dosage of three infusions a day. After 2 days, the neutrophil counts and CRP levels started to decrease drastically, reaching basal values, and the two blood culture sets performed resulted negative. The patient no longer had fever, and the opioid patch was removed as he no longer felt any pain. A total body CT scan was performed before dismission, showing successful surgical site debridement with no residual collections (Figure 4).

## 3. Discussion

Since its pathogenic emergence nocardiosis has been characterized as a disease primarily affecting patients with weakened immune systems. This is due to the multitude of cases linked to comorbidities such as Hodgkin's lymphoma, AIDS, Cushing's syndrome, various forms of cancer, the use of drugs that suppress the immune system, lung diseases, chronic kidney diseases, and more. However, recent epidemiological research indicates that not all individuals diagnosed with nocardiosis have concurrent diseases or identifiable risk factors [4–11]. This case report emphasizes an exceptionally rare instance of *N. kroppenstedtii* infection, which also exhibits unusual localizations. It also provides the first account of *N. kroppenstedtii* being identified in drainage fluid and the first recorded infection of this pathogen of extreme rarity in a patient with a healthy immune system. *Nocardia kroppenstedtii* is known for causing severe invasive infections that can be life-threatening. Since its initial identification in 2014, only six cases have been reported in medical literature including this paper, with the majority resulting in fatal outcomes [12, 17–20] (Table 2). This case also marks the first time that MALDI-TOF has been successfully used in diagnosing *Nocardia kroppenstedtii*.

Current guidelines for treating nocardiosis suggest starting empirical therapy with TMP-SMX or alternative medications such as linezolid and amikacin. For more severe infections, a combination therapy of TMP-SMX and third-generation cephalosporins or carbapenems is recommended.

Antibiotic susceptibility testing confirmed that TMP-SMX and tigecycline were the most effective treatments, aligning with existing guidelines while both cephalosporins and carbapenems showed resistance. Interestingly, this particular strain presented a decreased susceptibility to meropenem.

## 4. Conclusion

The positive outcome of the patient's surgical and clinical treatment, facilitated by interdepartmental collaboration, underscores the significance of a multidisciplinary strategy. This strategy is crucial within the framework of antimicrobial diagnostic stewardship across laboratories and hospital units. It enhances both the treatment process and patient results in infection cases. This is particularly true when the infections are caused by exceptionally uncommon pathogens, as demonstrated in this case report.

## Figures and Tables

**Table 1:** Results of antimicrobial susceptibility testing (AST).

Drugs	MIC (mg/L)	Susceptibility
Amikacin	0.75	S
Ampicillin	8	R
Ampicillin/sulbactam	24	R
Cefotaxime	∗	R
Ciprofloxacin	∗	R
Gentamicin	∗	R
Linezolid	∗	S
Meropenem	∗	R
Meropenem/vaborbactam	3	S
Minocycline	∗	R
Tigecycline	0.25	S
Tobramycine	∗	R
Trimethoprim/sulfamethoxazole	1	S

*Note:* The table shows the antibacterial susceptibilities profiles of *N. kroppenstedtii* with the relative MIC and disk diffusion values, determined by referring to EUCAST guidelines 203 v. 13.1. S = susceptible; R = resistant. ∗ = disk diffusion.

**Table 2:** Details on the demographic, clinical history, and microbiological samples related to cases of *N. kroppenstedtii* isolation.

Author and reference year	Age	Gender	Factor of predisposition	Sample in which *N. kroppenstedtii* was isolated
Jones et al. (2014)	Not reported	Not reported	Lung transplant	Alveolar broncholavage
Majeed et al. (2017)	72	Male	Mantle cell lymphoma	Blood
Venkat et al. (2019)	Not reported	Female	Previous treatment for several malignancies	Blood
Tay et al. (2021)	78	Not reported	Immunosuppressed due to autoimmune hemolytic anemia	Spinal vertebral abscess
Xing et al. (2023)	71	Female	Still's disease	Alveolar broncholavage, blood, pleura effusion
Presented scenario	69	Male	Unknown, immunocompetent	Surgical drainage fluids

## Data Availability

All data generated or analyzed during this study are included in this article.
